# Author Correction: Chemically-defined and scalable culture system for intestinal stem cells derived from human intestinal organoids

**DOI:** 10.1038/s41467-026-72959-8

**Published:** 2026-05-08

**Authors:** Ohman Kwon, Hana Lee, Jaeeun Jung, Ye Seul Son, Sojeong Jeon, Won Dong Yu, Naeun Son, Kwang Bo Jung, Eunho Choi, In-Chul Lee, Hyung-Jun Kwon, Chuna Kim, Mi-Ok Lee, Hyun-Soo Cho, Dae Soo Kim, Mi-Young Son

**Affiliations:** 1 Korea Research Institute of Bioscience and Biotechnology (KRIBB), Daejeon, 34141 Republic of Korea; 2 KRIBB School of Bioscience, Korea University of Science and Technology (UST), Daejeon, 34113 Republic of Korea; 3 Korea Research Institute of Bioscience and Biotechnology (KRIBB), Jeongeup, 56212 Republic of Korea; 4 KRIBB, Korea Preclinical Evaluation Center, Jeongeup, 56212 Republic of Korea; 5 KRIBB, Aging Convergence Research Center, Daejeon, 34141 Republic of Korea; 6 Department of Biological Science, Sungkyunkwan University, Suwon, 16419 Republic of Korea

**Keywords:** Intestinal stem cells, Gastrointestinal models, Stem-cell biotechnology, Intestinal stem cells

Correction to: *Nature Communications* 10.1038/s41467-024-45103-7, published online 27 January 2024

In this article the author’s name Won Dong Yu was incorrectly written as Won Dong Yoo. The original article has been updated.

