# Supplementary material for: Chemically-defined and scalable culture system for intestinal stem cells derived from human intestinal organoids
Source: Nat Commun. 2024 Jan 27;15:799. doi: 10.1038/s41467-024-45103-7 (PMC10821882; doi:10.1038/s41467-024-45103-7)
Supplement: Supplementary file 1 — SUPPLEMENTARY INFORMATION [file 41467_2024_45103_MOESM1_ESM.pdf]

SUPPLEMENTARY INFORMATION

Chemically-defined and scalable culture system for intestinal stem cells derived from human intestinal organoids

Authors:

Ohman Kwon[1], Hana Lee[1], Jaeeun Jung[1], Ye Seul Son[1], Sojeong Jeon[1], Won Dong Yoo[1,2], Naeun Son[1,2], Kwang Bo Jung[1], Eunho Choi[1,2], In-Chul Lee[3,4], Hyung-Jun Kwon[3,4], Chuna Kim[1,2,5], Mi-Ok Lee[1,2], Hyun-Soo Cho[1,2,6], Dae Soo Kim[1,2], and Mi-Young Son[1,2,6*]

Affiliations:

[1]Korea Research Institute of Bioscience and Biotechnology (KRIBB), Daejeon, 34141, Republic of Korea
[2]KRIBB School of Bioscience, Korea University of Science and Technology (UST), Daejeon, 34113, Republic of Korea;
[3]Korea Research Institute of Bioscience and Biotechnology (KRIBB), Jeongeup, 56212, Republic of Korea
[4]Korea Preclinical Evaluation Center, Jeongeup, 56212, Republic of Korea
[5]Aging Convergence Research Center, Daejeon, 34141, Republic of Korea
[6]Department of Biological Science, Sungkyunkwan University, Suwon, 16419, Republic of Korea

***Corresponding author:**

Mi-Young Son (E-mail: myson@kribb.re.kr; Tel: 82-42-860-4426; Fax 82-42-860-4608)

Supplementary Figures 1-9

Supplementary Table 1-3

Supplementary Figure 1

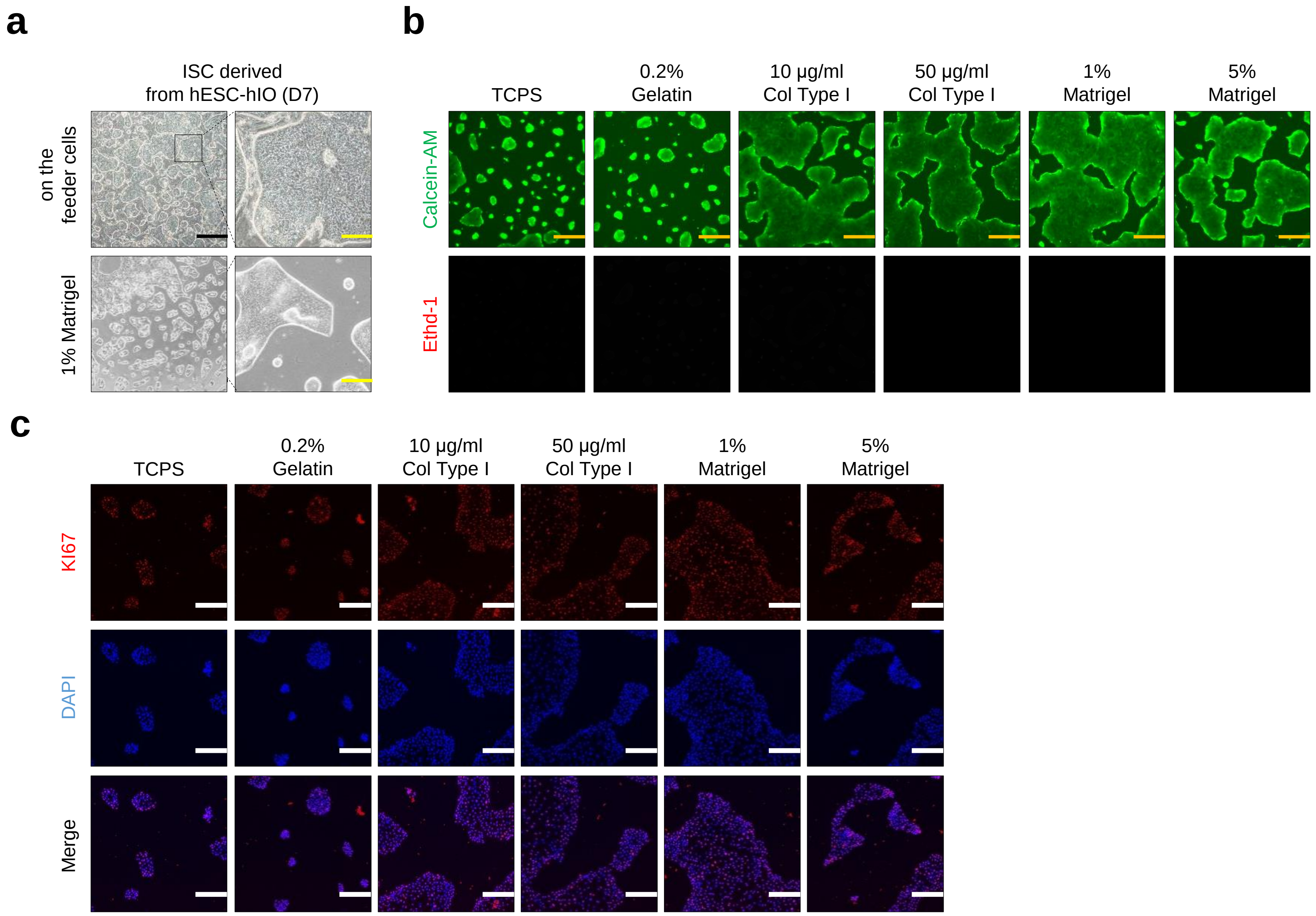

Supplementary Figure 1. Establishment of ISC$^{3D\text{-}hIO}$ culture system. a. Morphologies of ISC$^{3D\text{-}hIO}$ generated from hESC-derived hIOs on the feeder or 1% Matrigel-coated plate at day 7. Black scale bar: 200 µm. Yellow scale bar: 50 µm. **b.** Live (Calcein-AM)/Dead (EthD-1) analysis of ISC$^{3D\text{-}hIO}$, Orange scale bar: 200 µm. **c.** Immunofluorescence images of KI67, White scale bar: 100 µm.

Supplementary Figure 2

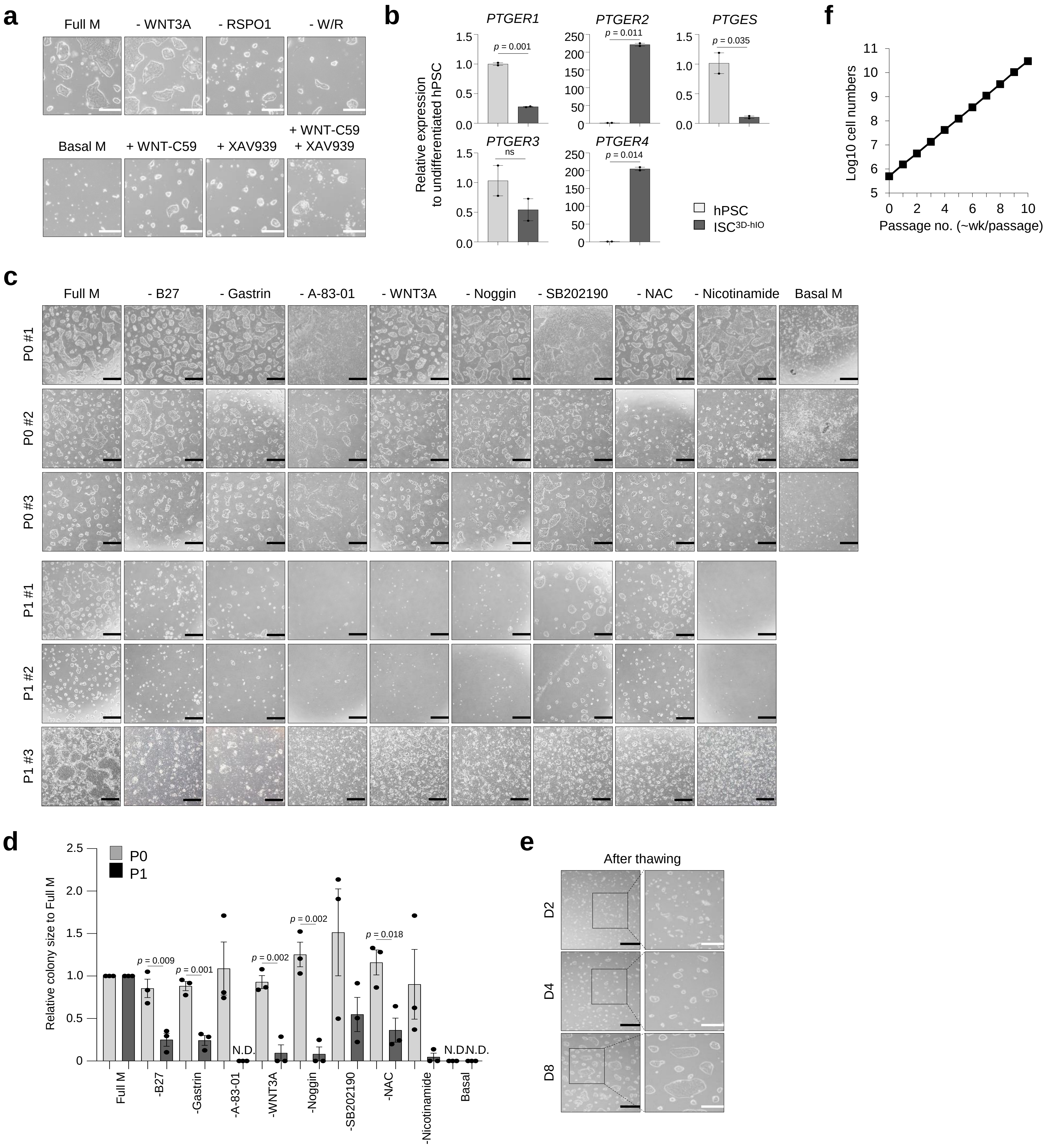

Supplementary Figure 2. Optimisation of the ISC$^{\text{3D-hIO}}$ full growth media composition. a. Representative images of ISC$^{\text{3D-hIO}}$ in Full M, depletion of WNT3A, RSPO1, or WNT3A/RSPO1, treatment with WNT-C59 (1 μM), XAV-939 (1 μM), or WNT-C59/XAV-939, and Basal M. White scale bar: 100 μm. **b.** Relative expression of PGE2 receptors (*PTGER1-4*) and synthesizing enzyme (*PTGES*) in ISC$^{\text{3D-hIO}}$. Data represents the mean ± SEM (n = 2 biological samples), and a two-tailed t-test was applied to measure p values between hPSC and ISC$^{\text{3D-hIO}}$. **c.** Representative images of ISC$^{\text{3D-hIO}}$ colonies with depletion of a single component from Full M at P0 and P1. Black scale bar: 200 μm. White scale bar: 100 μm. **d.** Relative ISC$^{\text{3D-hIO}}$ colony size to Full M. Data represents the mean ± SEM (n = 3 biological samples with 5 technical replicates), and a two-tailed t-test was applied to measure p values between the P0 cells and P1 cells. **e.** ISC$^{\text{3D-hIO}}$ morphologies at days 2, 4, and 8 after cell thawing. Black scale bar: 200 μm. White scale bar: 100 μm. **f.** Estimated cell numbers of ISC$^{\text{3D-hIO}}$ from passage number 0 to 10.

Supplementary Figure 3

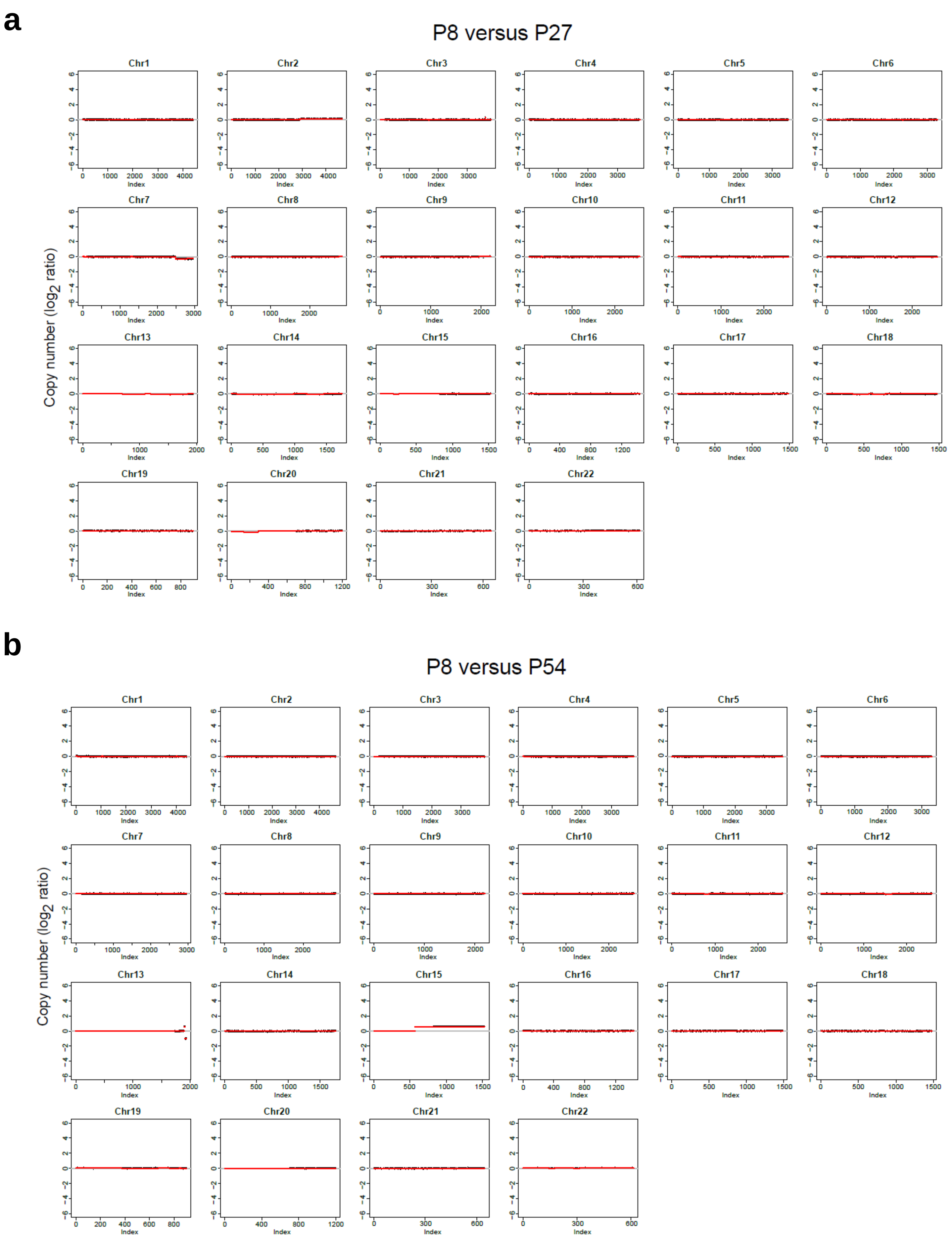

Supplementary Figure 3. Whole-genome profiling of copy number variation (CNV) using whole genome short-read sequencing data. a. CNV difference between p8 and p27 samples. **b.** CNV difference between p8 and p54 samples.

Supplementary Figure 4

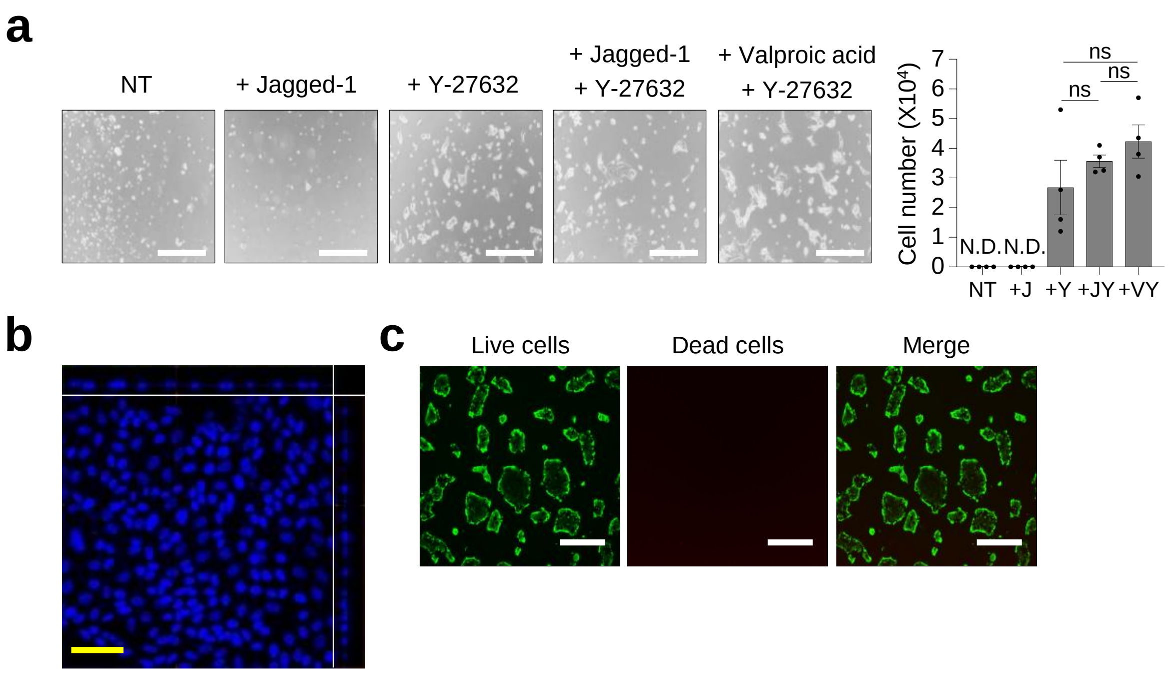

Supplementary Figure 4. Characterisation of the ISC$^{3D\text{-}hIO}$ monolayer. a. Cell attachment efficiency of ISC$^{3D\text{-}hIO}$ with treatment of Jagged-1 (1 µM), Y-27632 (10 µM), Valproic acid (1 mM), Jagged-1/Y-27632, and Jagged-1/Valproic acid. Error bars in the graph represent the mean ± SEM (n = 4 biological samples), and a two-tailed t-test was applied to measure p values among the cell treated with +Y, +JY, or +VY. **b.** Confocal image of the ISC$^{3D\text{-}hIO}$ monolayer with DAPI staining. **c.** Live/Dead analysis of ISC$^{3D\text{-}hIO}$ monolayer. White scale bar: 100 µm. Yellow scale bar: 50 µm.

Supplementary Figure 5

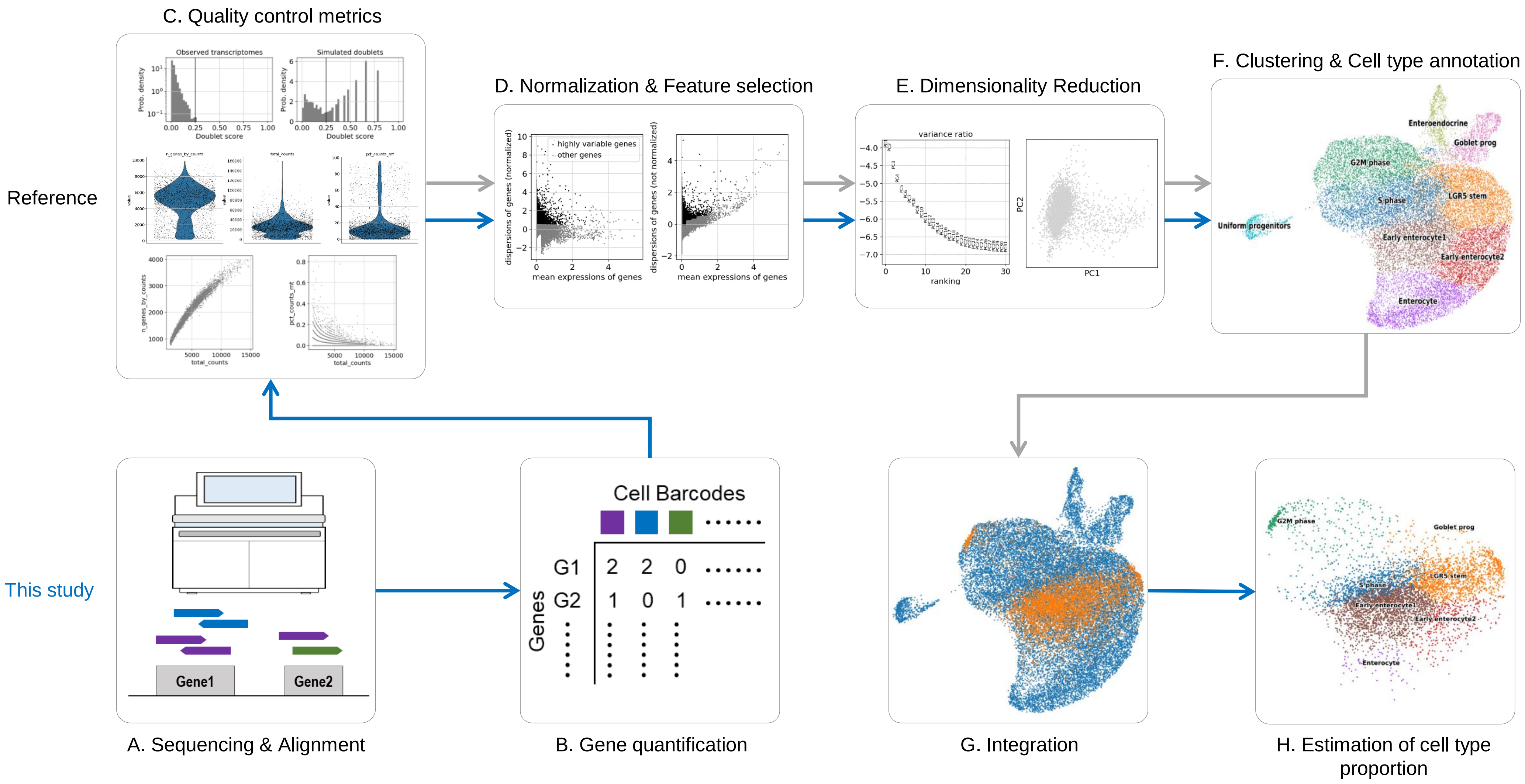

Supplementary Figure 5. An overview of scRNA-seq data processing workflow. Raw sequencing data were aligned using CellRanger (A) to generate a gene expression matrix (B). To integrate the scRNA-seq data with the published scRNA-seq dataset, confirm the quality of the reference scRNA-seq datasets generated by Elmentaite et al.[1, 2] (C). After normalizing expression values and removing batch effects from each dataset, select features (genes) that are consistently expressed (D). Perform dimensionality reduction using PCA and check for consistency between datasets (E). Then, conduct clustering and cell type identification on the integrated data (F). Compare gene expression patterns in our data and reference data to identify significant differences (G). Visually compare gene expression between cell types under different conditions, and calculate cell proportions for our scRNA-seq data (H).

Supplementary Figure 6

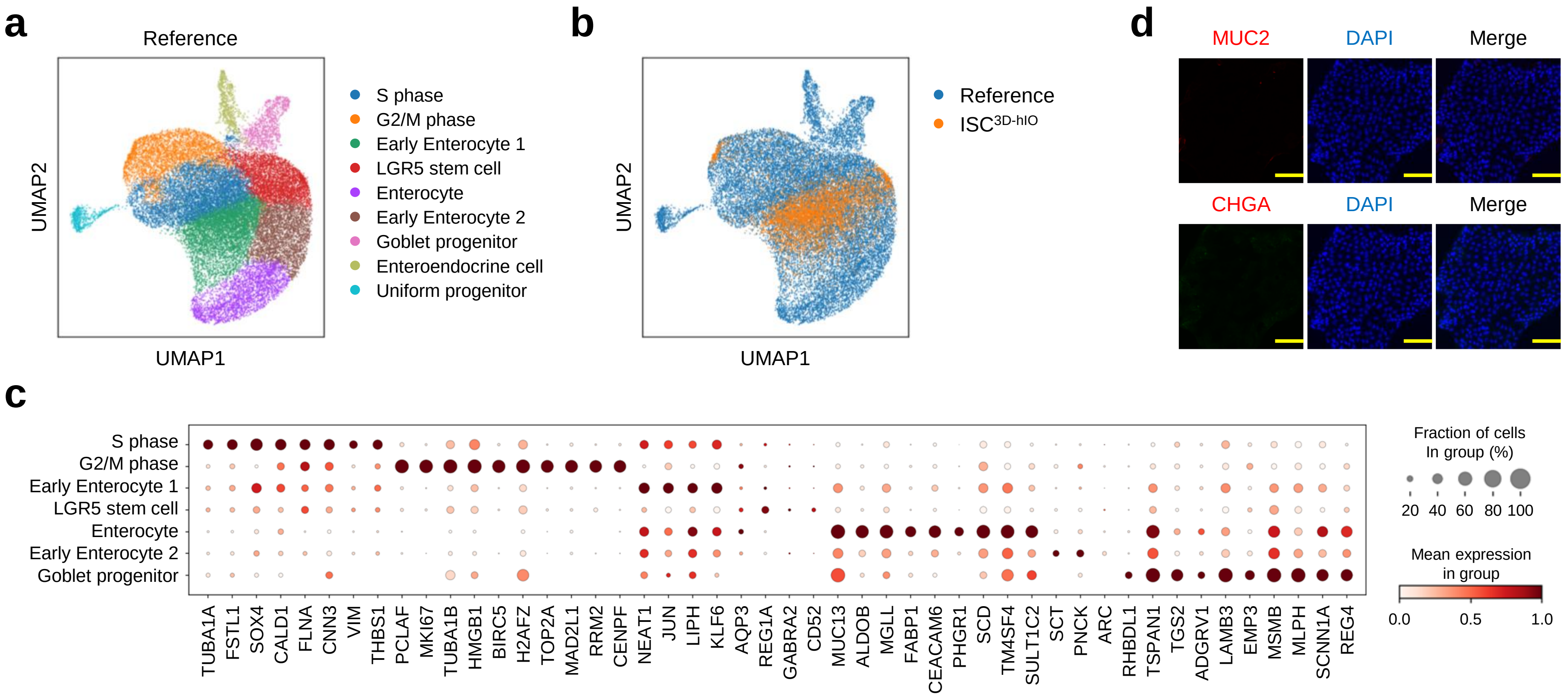

Supplementary Figure 6. Single-cell profiling of the ISC$^{3D\text{-}hIO}$. a. UMAP plots of ISC$^{3D\text{-}hIO}$ and reference datasets[1, 2] were separated into nine groups: S-phase (blue), G2/M phase (orange), Early enterocyte 1 (green), LGR5 stem cell (red), Enterocyte (purple), Early enterocyte 2 (brown), Goblet progenitor (pink), Enteroendocrine cell (dark khaki), and Uniform progenitor (cyan). **b.** UMAP plots visualised predicted cell types of ISC$^{3D\text{-}hIO}$ by overlaid with reference datasets: reference dataset [1, 2] (blue) and ISC$^{3D\text{-}hIO}$ (orange). **c.** Dot plot of expressed marker genes used for cell type annotation. **d.** Immunofluorescence images of goblet cell marker (*MUC2)* and enteroendocrine cell marker (*CHGA*). Yellow scale bar: 50 μm.

Supplementary Figure 7

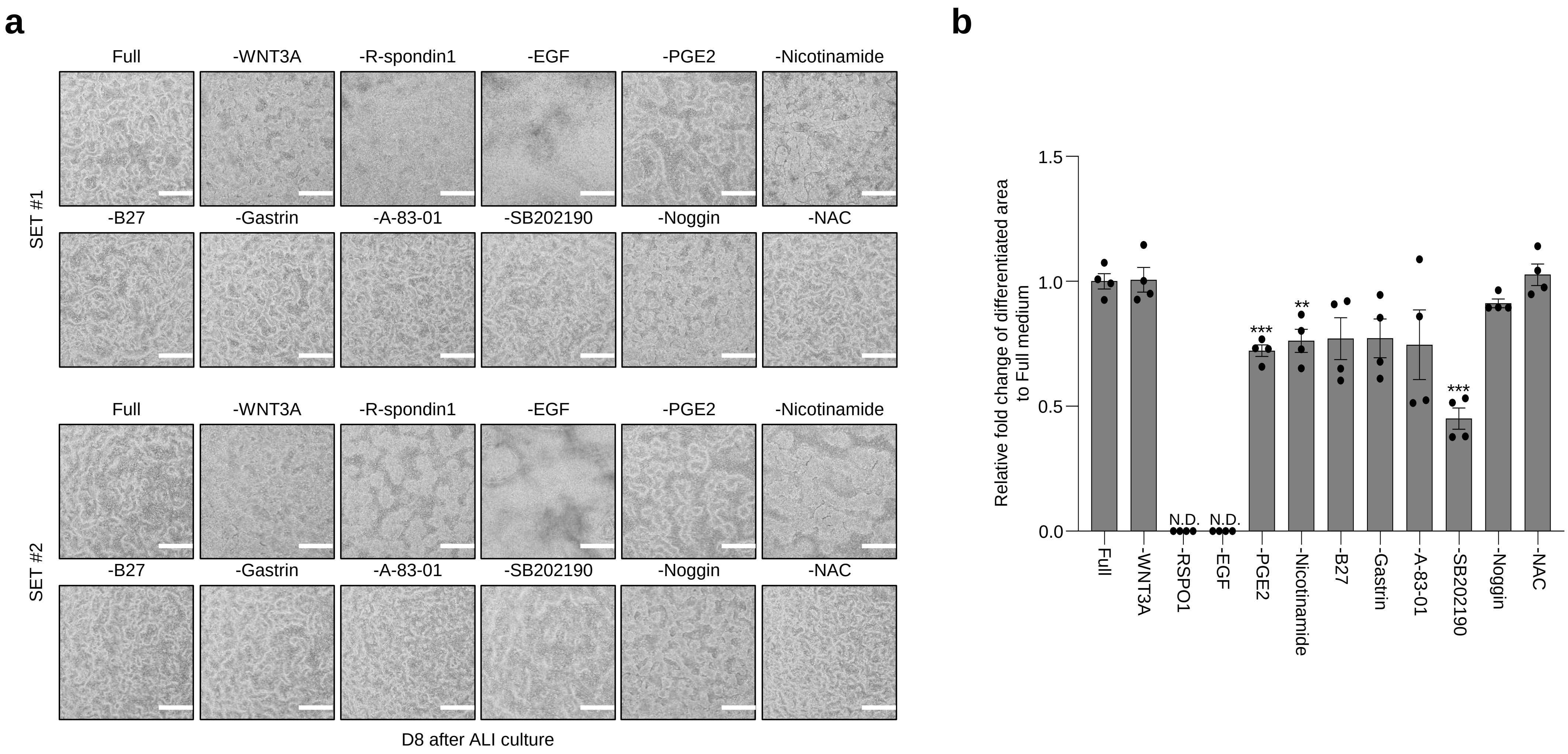

Supplementary Figure 7. The effect of single component depletion from the differentiation media on the ALI-differentiated cells. a. Morphologies of ALI-differentiated cells grown in single component depleted conditions at day 8 after air exposure. White scale bar: 100 μm. **b.** Quantification analysis of 2.5D intestinal epithelium in full growth medium or each component depleted medium. Data represent the mean ± SEM (n = 4 biological samples), and a two-tailed t-test was applied to measure p values between the control cells (Full) and cell grown in the single factor depleted conditions. $p < 0.001$ (Full vs -PGE_2), $p = 0.007$ (Full vs Nicotinamide), and $p < 0.001$ (Full vs –SB202190).

Supplementary Figure 8

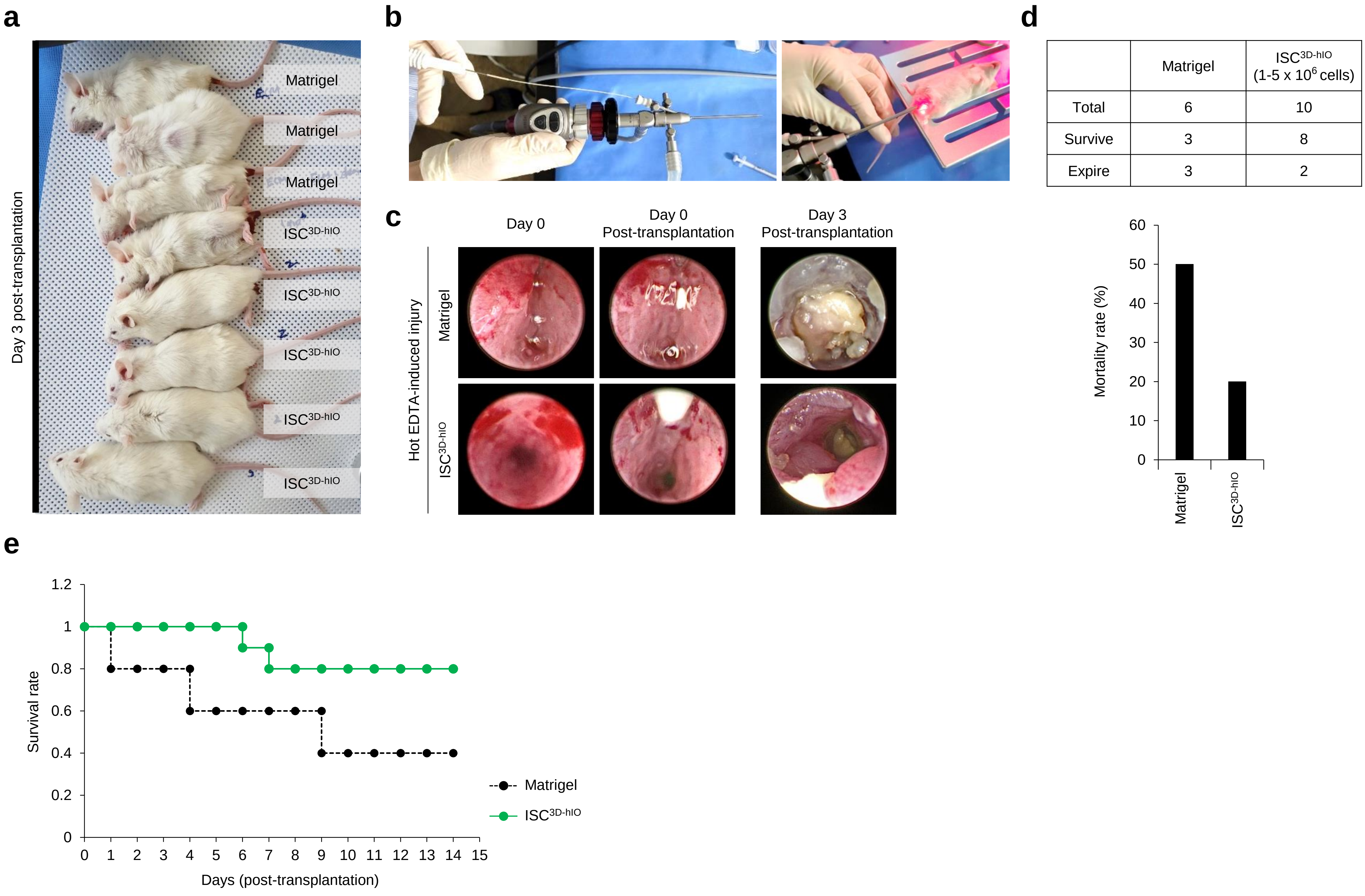

	Matrigel	$ISC^{3D\text{-}hIO}$ ($1\text{-}5 \times 10^6$ cells)
Total	6	10
Survive	3	8
Expire	3	2

Supplementary Figure 8. Xenograft of $ISC^{3D\text{-}hIO}$ in an EDTA-induced epithelial injury model. a. Representative mice images at day 3 post-transplantation. **b.** Pictures of the colonoscope equipment with a needle and the colonoscopy-guided transplantation into the mouse. **c.** Colonoscopy images of $ISC^{3D\text{-}hIO}$ transplantation with the needle at days 0 and 3 post-transplantation. **d.** A summary of experimental mice used and the mortality rate. **e.** Kaplan-Meier plot for survival rate analysis. (Matrigel, n=6; $ISC^{3D\text{-}hIO}$, n=10)

Supplementary Figure 9

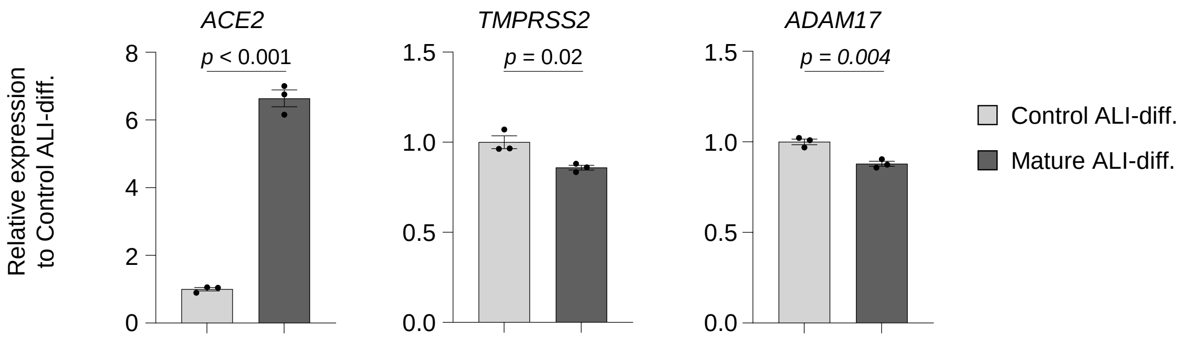

Supplementary Figure 9. Gene expression levels associated with SARS-CoV-19 entry in RNA-seq data. Relative RNA expression levels of *ACE2*, *TMPRSS*, and *ADAM17* in Mature ALI-diff. compared to Control ALI-diff. Data represent the mean ± SEM (n = 3 biological samples), and a two-tailed t-test was applied to measure p values between control ALI-diff. and mature ALI-diff.

Supplementary Table 1. Ingredients of ISC^{3D-hIO} growth medium.

		Supplier	Cat NO.	Solvent	Stock Conc.	Final Conc.
Basal	adv DMEM/F12	Gibco	12634	-	-	-
	HEPES	Invitrogen	15630-080	-	1 M	15 mM
	L-Gln (or GlutaMAX)	Invitrogen	25030081	-	200 mM	2 mM
	B27	Invitrogen	12587-010	-	50X	1X
	N-acetylcysteine	Sigma-Aldrich	A9165-5G	DW	500 mM	1 mM
	Penicillin/Streptomycin	Invitrogen	15140-122	-	10000 U/ml	100 U/ml
	Nicotinamide	Sigma-Aldrich	N0636	DW	1 M	10 mM
Supplement	EGF	R&D	236-EG-01M	1% BSA/PBS	500 μg/ml	100 ng/ml
	Noggin	R&D	6057-NG-100	1% BSA/PBS	100 μg/ml	100 ng/ml
	R-spondin	Peprotech	120-38	1% BSA/PBS	500 μg/ml	500 ng/ml
	[Leu15]-Gastrin I	Sigma-aldrich	G9145-1MG	1% BSA/PBS	100 μM	10 nM
	human rWnt3a	R&D	5036-WN-500	PBS	250 μg/ml	100 ng/ml
	A-83-01	Tocris	2939	DMSO	5 mM	500 nM
	SB202190	Sigma-aldrich	S7067	DMSO	30 mM	10 μM
	Prostaglandin E2	Sigma-aldrich	P0409	DW	5 mM	2.5 μM
	Jagged-1 (Transfer medium only)	Anaspc	AS-61298	DW	500 μM	1 μM
	Y-27632 (Transfer medium only)	Tocris	1254	DW	10 mM	10 μM

Supplementary Table 2. Antibodies used in this study.

Antibodies	Catalog No.	Company	Dilution
anti-LDHB	PA5-96736	Thermo Scientific	1:200 for IF
anti-EIF3E	NBP1-84869	NOVUS	1:100 for IF
anti-SOX9	sc-166505	Santa Cruz	1:100 for IF
anti-KI67	556003	BD	1:100 for IF
anti-CD44	ab6124	abcam	1:200 for IF
anti-KRT20	ab76126	abcam	1:100 for IF
anti-Villin1	sc-7672	Santa Cruz	1:50 for IF
anti-Mucin2	sc-7314	Santa Cruz	1:50 for IF
anti-Lysozyme	ab76784	abcam	1:200 for IF
anti-Chromogranin A	MA5-14536	Thermo Scientific	1:100 for IF
anti-ECAD	AF648	R&D systems	1:200 for IF
anti-FABP1	13368	Cell signaling Technology	1:100 for IF
anti-Cytokeratin Pure CAM 5.2	349205	BD Biosciences	25 μg/mL for IF
anti-ACE2	AF933	R&D systems	1:100 for IF

Supplementary Table 3. Primers used in this study.

Target gene	Primer (Forward)	Primer (Reverse)
GAPDH	GAAGGTGAAGGTCGGAGTC	GAAGATGGTGATGGGATTTC
LGR5	TGCTCTTCACCAACTGCATC	CTCAGGCTCACCAGATCCTC
CD44	CCAGAAGGAACAGTGGTTTGGC	ACTGTCCTCTGGGCTTGGTGTT
SOX9	GTACCCGCACTTGCACAAC	TCTCGCTCTCGTTCAGAAGTC
LRIG1	GACCCTTTCTGACCGACAA	CGCTTTCCACGGCTCTTT
LYZ	AAAACCCCAGGAGCAGTTAAT	CAACCCTCTTTGCACAAGCT
MKL67	TGACCCTGATGAGAAAGCTCAA	CCCTGAGCAACACTGTCTTTT
AXIN2	GAGTGGACTTGTGCCGACTTCA	GGTGGCTGGTGCAAAGACATAG
CTNNB	TCTGAGGACAAGCCACAAGATTACA	TGGGCACCAATATCAAGTCCAA
ASCL2	CGTGAAGCTGGTGAACTTGG	GGATGTACTCCACGGCTGAG
OLFM4	ACCTTTCCCGTGGACAGAGT	TGGACATATTCCCTCACTTTGGA
VIL1	AGCCAGATCACTGCTGAGGT	TGGACAGGTGTTCCTCCTTC
ECAD	GTCACTGACACCAACGATAATCCT	TTTCAGTGTGGTGATTACGACGTTA
FABP1	GGAGGAATGTGAGCTGGAGACA	TATGTCGCCGTTGAGTTCGGTC
KRT20	TGGCCTACACAAGCATCTGG	TAACTGGCTGCTGTAACGGG
LCT	GGCAGTCTGGGAGTTTTAGG	ATGCCAAAATGAGGCAAGTC
MUC2	TGTAGGCATCGCTCTTCTCA	GACACCATCTACCTCACCCG
CHGA	TGACCTCAACGATGCATTTC	CTGTCCTGGCTCTTCTGCTC
AKR1B15	GAGGACCTGTTCATCGTCAGCA	CGTCCAGATAGCTCAGCTTCAG
DHRS11	CACCAGTGGTTGGAAGGACATG	CATCGTCCACATTCCGCTCCTT
GALNT4	GTCAAGAAGGCTCTCAGACCTC	GTTCATCCTCGTTGAGCTGGAG
GALNT5	CCAGTGGATAGAGCCATTGAAGA	TCTCAGGAGAGTGGACCACACT
DHRS3	GGGCACTGAGTGCCATTACTTC	CGGCATTGTTCACCAGGATGGT
RDH10	GGCATCACCTTCTGGAATGTCC	CCAGCATCGTAGGAAGAAAAGCC
AADAC	CGGTATTTCTGGAGATAGTGCAG	TCAAGAGGCTGAAGGGCAGGAT
NR1I2	GCTGTCCTACTGCTTGGAAGAC	CTGCATCAGCACATACTCCTCC
SULTE1	CTGCATCAGCACATACTCCTCC	CCAGGATTTGGATGACCAGCCA
DUOX2	CCAGGATTTGGATGACCAGCCA	GTCCTTGGAGAGGAAGCCATTC
SLC6A20	CAGCGAGATGTTCCCGCAAATC	GCCTCTGTGTAGACGATGAATGC
SLC43A1	GATGCTGGAGTACCTTGTGACTG	CAGGTGAGAAGGCACAACAGCT
DPP4	CAAATTGAAGCAGCCAGACA	GGAGTTGGGAGACCCATGTA
DEFA5	CCTTTGCAGGAAATGGACTC	GGACTCACGGGTAGCACAAC
ZO-1	TGTGAGTCCTTCAGCTGTGGAA	GGAACTCAACACACCATTG
OCLD	CATTGCCATCTTTGCCTGTG	AGCCATAACCATAGCCATAGC
CLDN1	CCCAGTCAATGCCAGGTACG	GGGCCTTGGTGTTGGGTAAG
CLDN3	CAGGCTACGACCGCAAGGAC	GGTGGTGGTGGTGGTGTTGG
CLDN5	GCAGCCCCTGTGAAGATTGA	GTCTCTGGCAAAAAGCGGTG
ACE2	TCCATTGGTCTTCTGTCACCCG	AGACCATCCACCTCCACTTCTC
PTGES	GAGGATGCCCTGAGACACGGA	CCAGAAAGGAGTAGACGAAGCC
PTGER1	ATGGTGGTGTCGTGCATCT	CGCTGCAGGGAGGTAGAG
PTGER2	CCACCTCATTCTCCTGGCTA	AGGTCCCATTTTTCCTTTCG
PTGER3	ATCATGTGCGTGCTGTCG	TGCAGTGCTCAACTGATGTCT
PTGER4	CTCCCTGGTGGTGCTCAT	GGCTGATATAACTGGTTGACGA
N gene	GACCCCAAA ATCAGCGAAAT	TCTGGTTACTGCCAGTTGAATCTG
E gene	AGCAGTACGCACACAATCG	TTCGGAAGAGACAGGTACGTTA
RdRP	CTCCTCTAGTGGCGGCTATT	AGAATAGAGCTCGCACCGTA

References

1. Elmentaite, R. et al. Single-Cell Sequencing of Developing Human Gut Reveals Transcriptional Links to Childhood Crohn's Disease. Dev Cell 55, 771-783 e775 (2020).
2. Elmentaite, R. et al. Cells of the human intestinal tract mapped across space and time. Nature 597, 250-255 (2021).
