# Supplementary material for: Chemically-defined and scalable culture system for intestinal stem cells derived from human intestinal organoids
Source: Nat Commun. 2024 Jan 27;15:799. doi: 10.1038/s41467-024-45103-7 (PMC10821882; doi:10.1038/s41467-024-45103-7)
Supplement: Supplementary file 3 — Reporting Summary [file 41467_2024_45103_MOESM3_ESM.pdf]

nature portfolio

Corresponding author(s): Son, Mi-Young

Last updated by author(s): Nov 30, 2023

Reporting Summary

Nature Portfolio wishes to improve the reproducibility of the work that we publish. This form provides structure for consistency and transparency in reporting. For further information on Nature Portfolio policies, see our Editorial Policies and the Editorial Policy Checklist.

Statistics

For all statistical analyses, confirm that the following items are present in the figure legend, table legend, main text, or Methods section.

n/a	Confirmed	
☐	☒	The exact sample size (*n*) for each experimental group/condition, given as a discrete number and unit of measurement
☐	☒	A statement on whether measurements were taken from distinct samples or whether the same sample was measured repeatedly
☐	☒	The statistical test(s) used AND whether they are one- or two-sided<br>*Only common tests should be described solely by name; describe more complex techniques in the Methods section.*
☐	☒	A description of all covariates tested
☐	☒	A description of any assumptions or corrections, such as tests of normality and adjustment for multiple comparisons
☐	☒	A full description of the statistical parameters including central tendency (e.g. means) or other basic estimates (e.g. regression coefficient) AND variation (e.g. standard deviation) or associated estimates of uncertainty (e.g. confidence intervals)
☐	☒	For null hypothesis testing, the test statistic (e.g. *F*, *t*, *r*) with confidence intervals, effect sizes, degrees of freedom and *P* value noted<br>*Give P values as exact values whenever suitable.*
☒	☐	For Bayesian analysis, information on the choice of priors and Markov chain Monte Carlo settings
☐	☒	For hierarchical and complex designs, identification of the appropriate level for tests and full reporting of outcomes
☐	☒	Estimates of effect sizes (e.g. Cohen's *d*, Pearson's *r*), indicating how they were calculated

Our web collection on statistics for biologists contains articles on many of the points above.

Software and code

Policy information about availability of computer code

Data collection	For scRNA-seq data, libraries were generated using the Chromium Next GEM Single Cell 3' reagent kit Ver. 3.1 (10X genomics) according to the manufacturer's protocol. And then, the libraries were sequenced in multiplex on a Novaseq 6000 sequencer (Illumina) to produce, on average, a minimum of 60,000 reads per single-cell.<br><br>For bulk RNA-seq data, libraries were prepared using Illumina TruSeq library preparation according to the manufacturer’s specifications. And, RNA sequencing was performed on an Illumina HiSeq2500 following the standard Illumina RNA-Seq protocol, with a read length of 2×100 bases.
Data analysis	For scRNA-seq data analysis<br>10X Genomics software CellRanger Ver and 3.1<br>Scanpy package Ver1.8<br><br>For bulk RNA-seq data analysis<br>Agilent 2100 Bioanalyzer system<br>NGSQCToolkit Ver. 2.3.3<br>Cutadapt Ver. 1.18<br>Sickle Ver. 1.33<br>HISAT2 Ver. 2.0.5<br>StringTie Ver. 2.1.0

For the immunofluorescence image analysis
Image J 1.53e

For manuscripts utilizing custom algorithms or software that are central to the research but not yet described in published literature, software must be made available to editors and reviewers. We strongly encourage code deposition in a community repository (e.g. GitHub). See the Nature Portfolio guidelines for submitting code & software for further information.

Data

Policy information about availability of data

All manuscripts must include a data availability statement. This statement should provide the following information, where applicable:

- Accession codes, unique identifiers, or web links for publicly available datasets
- A description of any restrictions on data availability
- For clinical datasets or third party data, please ensure that the statement adheres to our policy

Data are provided in the main manuscript, supplementary materials. And, scRNA-seq data and bulk RNA-seq data are uploaded in the Gene Expression Omnibus (GSE219018).

Research involving human participants, their data, or biological material

Policy information about studies with human participants or human data. See also policy information about sex, gender (identity/presentation), and sexual orientation and race, ethnicity and racism.

Reporting on sex and gender	N/A. No human subjects are involved in this study.
Reporting on race, ethnicity, or other socially relevant groupings	N/A.
Population characteristics	N/A.
Recruitment	N/A.
Ethics oversight	N/A.

Note that full information on the approval of the study protocol must also be provided in the manuscript.

Field-specific reporting

Please select the one below that is the best fit for your research. If you are not sure, read the appropriate sections before making your selection.

☒ Life sciences ☐ Behavioural & social sciences ☐ Ecological, evolutionary & environmental sciences

For a reference copy of the document with all sections, see nature.com/documents/nr-reporting-summary-flat.pdf

Life sciences study design

All studies must disclose on these points even when the disclosure is negative.

Sample size	No statistical method was used to predetermine sample size. Sample sizes were determined based on whether data is reproducible in independent sets of experiments,
Data exclusions	No data was excluded.
Replication	Experiments were performed in triplicated unless specified.
Randomization	sample were not randomized for this study.
Blinding	Binding was not applicable since no comparison between sample groups were made.

Reporting for specific materials, systems and methods

We require information from authors about some types of materials, experimental systems and methods used in many studies. Here, indicate whether each material, system or method listed is relevant to your study. If you are not sure if a list item applies to your research, read the appropriate section before selecting a response.

Materials & experimental systems

n/a	Involved in the study	
☐	☒	Antibodies
☐	☒	Eukaryotic cell lines
☒	☐	Palaeontology and archaeology
☐	☒	Animals and other organisms
☒	☐	Clinical data
☒	☐	Dual use research of concern
☒	☐	Plants

Methods

n/a	Involved in the study	
☒	☐	ChIP-seq
☒	☐	Flow cytometry
☒	☐	MRI-based neuroimaging

Antibodies

Antibodies used

anti-LDHB, Thermo Scientific, PA5-96736, Polyclonal, 1:200 for ICC
https://www.thermofisher.com/antibody/product/LDHB-Antibody-Polyclonal/PA5-96736

anti-EIF3E, NOVUS Biologicals, NBP1-84869, Polyclonal, 0.25-2 ug/ml for ICC
https://www.novusbio.com/products/eif3e-antibody_nbp1-84869

anti-SOX9, SANTA CRUZ Biotechnology, sc-166505, Monoclonal, 1:50 for ICC
https://www.scbt.com/p/sox-9-antibody-e-9

anti-KI67, BD Bioscience, 556003, Monoclonal
https://www.bdbiosciences.com/ko-kr/products/reagents/microscopy-imaging-reagents/immunofluorescence-reagents/purified-mouse-anti-ki-67.556003

anti-CD44, Abcam, ab6124, Monoclonal, 5 ug/ml for ICC
https://www.abcam.com/products/primary-antibodies/cd44-antibody-f10-44-2-ab6124.html

anti-KRT20, Abcam, ab76126, Monoclonal, 1:100 for ICC
https://www.abcam.com/products/primary-antibodies/cytokeratin-20-antibody-epr1622y-cytoskeleton-marker-ab76126.html

anti-Villin1, SANTA CRUZ Biotechnology, sc-7672, Polyclonal, 1:50 for ICC
https://www.scbt.com/p/villin-antibody-c-19

anti-Mucin2, SANTA CRUZ Biotechnology, sc-7314, Monoclonal, 1:50 for ICC
https://www.scbt.com/p/mucin-2-antibody-ccp58

anti-Lysozyme, Abcam, ab76784, Polyclonal, 1:100 for ICC
https://www.abcam.com/products/primary-antibodies/lysozyme-antibody-ab76784.html

anti-Chromogranin A, Thermo Scientific, MA5-14536, Monoclonal, 1:100 for ICC
https://www.thermofisher.com/antibody/product/Chromogranin-A-Antibody-clone-SP12-Monoclonal/MA5-14536

anti-ECAD, R&D Biosystems, AF648, Polyclonal, 5-15 ug/ml for ICC
https://www.rndsystems.com/products/human-mouse-e-cadherin-antibody_af648

anti-FABP1, Cell signaling Technology, 13368, Monoclonal, 1:50 for ICC
https://www.cellsignal.com/products/primary-antibodies/fabp1-d2a3x-xp-rabbit-mab/13368

anti-Cytokeratin Pure CAM5.2, BD Bioscience, 349205, Monoclonal
https://www.bdbiosciences.com/ko-kr/products/reagents/flow-cytometry-reagents/clinical-diagnostics/single-color-antibodies-asr-ivd-ce-ivd/anti-cytokeratin-purified.349205

anti-ACE2, R&D Biosystems, AF933, Polyclonal, 3-15 ug/ml for ICC
https://www.rndsystems.com/products/human-mouse-rat-hamster-ace-2-antibody_af933

Validation

We used only commersial antibodies which have been validated by the selling companies. Furthermore, the antibodies have been further validated in numerous published research papers citing the antibodies.

Eukaryotic cell lines

Policy information about cell lines and Sex and Gender in Research

Cell line source(s)	H9 hESCs were purchased from WiCell.<br>Human iPSCs were generated by our previous works.
Authentication	Not any wild animal was used in this study.
Mycoplasma contamination	Confirm that all cell lines regularly tested negative for mycoplasma comtamination.
Commonly misidentified lines<br>(See ICLAC register)	This study did not involve commoly misidentified lines.

Animals and other research organisms

Policy information about studies involving animals; ARRIVE guidelines recommended for reporting animal research, and Sex and Gender in Research

Laboratory animals	The male NIG (NOD/SCID deleted IL2Rg gene) aged 6-12 weeks was purchased from GHBIO, Daejeon, South Korea.
Wild animals	Not any wild animal was used in this study.
Reporting on sex	Gender and gender-based analyzes are not performed in the study. Referring to Sugimoto et al., 2018, who reported the intestinal epithelial injury model, male mice were used, and only changes by transplanted cells were observed regardless of sex.<br>The sex of animals used in experiments is reported in Figure Legends and Methods.
Field-collected samples	This study did not involve samples collected from the field.
Ethics oversight	The mouse experiments were performed with the approval of the Institutional Animal Care and Use Committee (IACUC) of KRIBB (approval number: KRIBB-AEC-21236).

Note that full information on the approval of the study protocol must also be provided in the manuscript.

Plants

Seed stocks	Not any plant sample was used in this study.
Novel plant genotypes	Not any plant sample was used in this study.
Authentication	Not any plant sample was used in this study.
